# A Fully-Sealed Carbon-Nanotube Cold-Cathode Terahertz Gyrotron

**DOI:** 10.1038/srep32936

**Published:** 2016-09-09

**Authors:** Xuesong Yuan, Weiwei Zhu, Yu Zhang, Ningsheng Xu, Yang Yan, Jianqiang Wu, Yan Shen, Jun Chen, Juncong She, Shaozhi Deng

**Affiliations:** 1 School of Physical Electronics,University of Electronic Science and Technology of China, Chengdu, 610054, China; 2 State Key Laboratory Optoelectronic Materials and Technologies, Guangdong Province Key Laboratory of Display Material and Technology, and School of Electronics and Information Technology, Sun Yat-sen University, Guangzhou, 510275, China

## Abstract

Gigahertz to terahertz radiation sources based on cold-cathode vacuum electron technology are pursued, because its unique characteristics of instant switch-on and power saving are important to military and space applications. Gigahertz gyrotron was reported using carbon nanotube (CNT) cold-cathode. It is reported here in first time that a fully-sealed CNT cold-cathode 0.22 THz-gyrotron is realized, typically with output power of 500 mW. To achieve this, we have studied mechanisms responsible for CNTs growth on curved shape metal surface, field emission from the sidewall of a CNT, and crystallized interface junction between CNT and substrate material. We have obtained uniform growth of CNTs on and direct growth from cone-cylinder stainless-steel electrode surface, and field emission from both tips and sidewalls of CNTs. It is essential for the success of a CNT terahertz gyrotron to have such high quality, high emitting performance CNTs. Also, we have developed a magnetic injection electron gun using CNT cold-cathode to exploit the advantages of such a conventional gun design, so that a large area emitting surface is utilized to deliver large current for electron beam. The results indicate that higher output power and higher radiation frequency terahertz gyrotron may be made using CNT cold-cathode electron gun.

High frequency vacuum electron radiation source is one important kind of devices that communication, satellites and spacecraft rely on widely. At present, the commercial vacuum electron radiation sources are based on thermionic cathode electron sources that require heating with high working temperature, and can not be switched on instantly, and their electron beam is usually modulated in an extended interaction line at downstream; this leads to large device size physically. The cold cathode is an ideal candidate for electron sources of high frequency radiation source device, which has the advantages of fast switch-on time and room working temperature, and can be minimized in the size of device and achieved functional integration of devices. In gigahertz frequencies, several prototypes of cold cathode devices have been demonstrated, such as travelling wave tubes^1^,^2^,^3^, gyrotrons^4^,^5^, klystrons^6^, and microwave diode etc^7^. In terahertz frequencies, the design and structure simulations of terahertz vacuum radiation source have been carried out^8^,^9^,^10^,^11^, but the experiments of the proposed terahertz cold-cathode devices have not been demonstrated as far as we know. Here we report in the first time the experimental results from a fully-sealed terahertz-gyrotron based on CNT cold-cathode. Our results thus move the frequency range from gigahertz region to terahertz region of cold-cathode high-frequency radiation sources.

Gyrotron, as so-called fast-wave vacuum electronic device, is based on the stimulated radiation of electron cyclotron resonance. It is one of the most important terahertz sources that can generate high power radiation in the millimeter-wave and terahertz frequency region. The range of operation beam current is very wide from tens of mA to hundreds of A in a gyrotron. The beam-wave interaction cavity of gyrotron is simple; it is a cylindrical waveguide.

The requirements of terahertz vacuum device on the cathode are high current, high current density and electron beam quality with good focus. Carbon nanotube (CNT) is the excellent field electron emitter; its field emission property with high current density has been reported by many authors including ourself^12^,^13^. How may CNTs be used in our present study? We decided to exploit the advantages of existing electron gun design, rather than having a new one. In this case, it is a conventional magnetic injection electron gun, where the cathode electrode is in cone-cylinder shape. Growth of CNTs on such a curved-shape metallic electrode has not been report before, and gives rise to both scientific and technological challenges. We have to develop a number of unique techniques so that uniform growth of carbon nanotubes on the surface of stainless-steel electrode in cone-cylinder shape is realized. But then how may one obtain high performance field emission CNTs on such a cold-cathode? Both minimizing electrical breakdown and increasing emission site are major challenges. Finally, to be able to deliver large current for forming electron beam is essential for a high output power gyrotron. Results below are from the studies of above problem.

## Results

### Terahertz gyrotron of CNT cold-cathode

**Figure 1:**
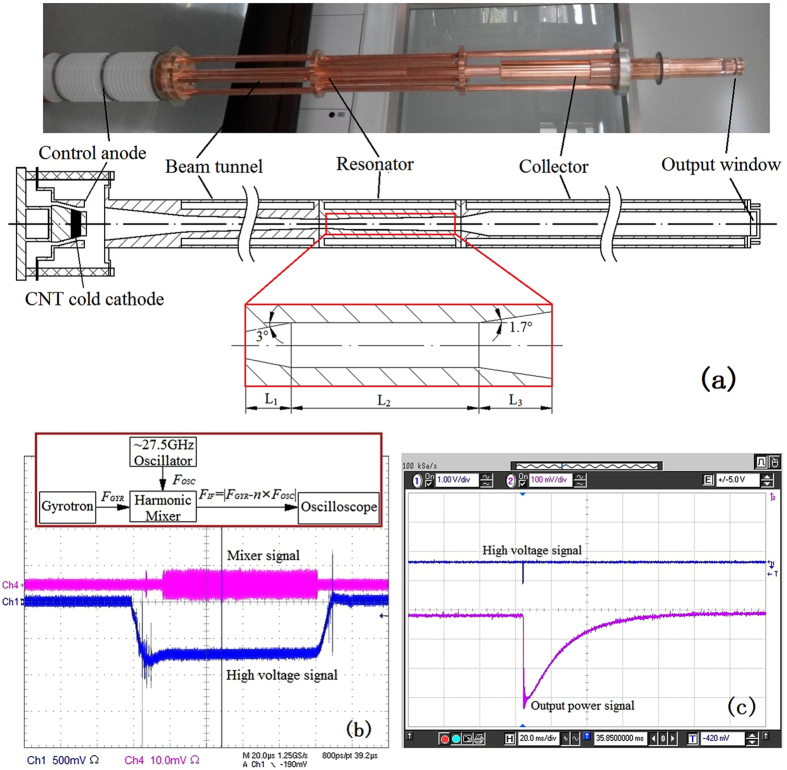
(**a**) Photo of the CNT cold-cathode terahertz gyrotron, and its cross-sectional illustration. (**b**,**c**) typical results of radiation output frequency and power measurement of the CNT cold-cathode gyrotron, where (**b**) output frequency signal results, inset: block diagram showing a harmonic mixer measurement arrangement, and (**c**) output power signal results.

Figure 1a is the photograph of the inner structure of our terahertz gyrotron device, accompanied with the schematic cross-section illustration. The gyrotron consists of a magnetic injection electron gun, beam-wave interaction resonator, collector and output window. The resonator is a three-section cylindrical waveguide cavity. The length *L*_1_ of the input taper with an angle of 3° is 9 mm, the midsection with a length *L*_2_ = 40 mm and a radius *R* = 2.2 mm is a cylindrical waveguide, and the length *L*_3_ of the output taper with an angle of 1.7° is 10 mm. The total quality factor *Q*_*T*_ of the resonator is about 12000, where *Q*_*T*_ = *Q*_*0*_*Q*_*d*_/(*Q*_*0*_ + *Q*_*d*_), while *Q*_*0*_ is the ohmic quality factor, and *Q*_*d*_ is the diffraction quality factor. Firstly, electrons are emitted from CNT cold-cathode under the action of the applied field between control anode and cold cathode. Because there are crossed electric and magnetic fields, electrons will do cyclotron motion along magnetic lines of force and form an electron beam, while they are accelerated by the anode into the beam tunnel. Here, magnetic field will gradually increase and electric field will not change, thus magnetic adiabatic compression will occur for the electron beam. In this process, the electron beam axial velocity will decrease, but its transverse velocity gradually increases. After the electron beam is compressed enough in the beam tunnel, it will enter the beam-wave interaction resonator, where it interacts with TE03 mode, generating electron cyclotron resonance maser.

Typical results of radiation output frequency and power measurement are given in Fig. 1b,c, respectively. With reference to the block diagram in Fig. 1b, the frequency of TE03 mode output from the gyrotron is measured by an 8^th^ harmonic mixer, which is also used as RF detector to measure the pulse width of output signal. From Fig. 1b, the pulse width of terahertz radiation signal is about 85 μs, shorter than that of high voltage signal (100 μs), because the latter is unstable within the first 15 μs due to capacitance effect. In frequency measurement, the local oscillator signal *F*_*OSC*_ is generated by an Analog Signal Generator (Agilent 8257D PSG), and intermediate frequency signal *F*_*IF*_ is detected by an Oscilloscope (Tektronix TDS 6604). At first, the frequency of *F*_*OSC*_ is set at 27.5125 GHz, and a 0.25 GHz intermediate frequency signal *F*_*IF*_ is detected by mixer. So the frequency of the radiation of the gyrotron is 0.21985 THz or 0.22035 THz, according to *F*_*GYR*_ = *8* × *F*_*OSC*_ ± *F*_*IF*_. Then, the frequency of *F*_*OSC*_ is set at 27.5375 GHz, and a 0.45 GHz intermediate frequency signal *F*_*IF*_ is obtained, giving 0.21985 THz or 0.22075 THz. Comparing with two detected results, the frequency of the CNT cold-cathode gyrotron may be determined to be 0.21985 THz, which agrees with the design.

Radiation output power measurements are made by using a calorimeter, which has the sensitivity allowing for detecting the terahertz wave energy in single shots at μJ level. Figure 1c shows a typical output power signal results. When one single pulse of high voltage signal is triggered, a thermal signal will be detected, because electromagnetic wave is absorbed by the calorimeter. By making use of another small power solid electron radiation with same operation frequency of 0.22 THz, a similar thermal signal will be obtained. Through calculating the thermal-signal integral changing with time, the energy of absorbed electromagnetic wave may be determined. Based on the energy conversion relationship thus obtained, the output power of the CNT cold-cathode gyrotron may be scaled. In the experimental process, output signal can be detected by applying different voltages to the cathode and the control anode, eg −36 kV for the cathode and −24 kV for the control anode, while anode is earthed and the magnetic field remains at 8.36 T. The power of CNT cold-cathode gyrotron is determined and is about 500 mW, when the high voltages for the cathode is −37 kV and −24.7 kV for the control anode.

### High performance electron-gun using the curved-shape CNT-stainless steel cold-cathode (curved-shape CNT-SS cathode)

**Figure 2:**
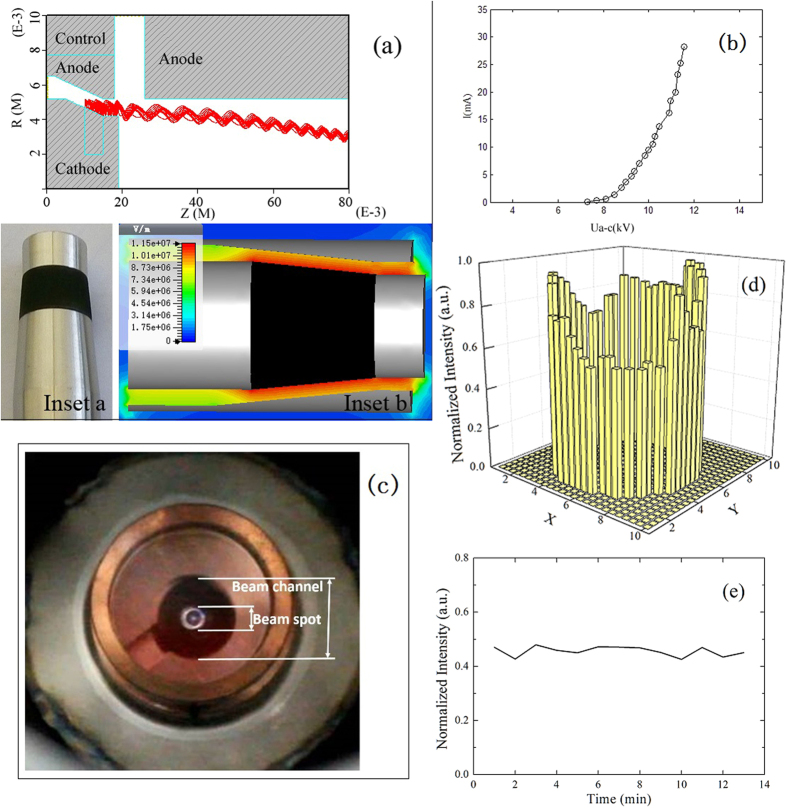
(**a**) The beam trajectories in a gun; inset a: photo of the curved-shape CNT-SS cathode, and inset b: electrostatic potential distribution on the axial section, (**b**) I-V characteristic of the electron gun: electron beam current vs voltage difference between control anode and cathode, (**c**) photo of electron beam spot, (**d**) histogram of brightness distribution along the ring spot, and (**e**) brightness stability of the ring spot in 14 minutes.

Before discussing the challenges we faced in the preparation of the CNT cold-cathode for the gyrotron device, we now give the advantages when such a device directly employs a coaxial magnetic injection electron gun with cone-cylinder cathode. As shown in Fig. 2a, the CNT cold-cathode is sandwiched between two stainless-steel cone cylinders where the vacuum gap is 1 mm and a gap of 0.5 mm for electrons passing through it along axis when an axial magnetic field is applied. In such a case, CNTs have to grow on the surface of stainless steel cathode electrode in cone-cylinder shape. We call it “curved-shape CNT-SS cathode”. Comparing with a flat cathode having control-grid, this kind of coaxial electron gun with cone-cylinder cathode has advantages. Firstly, the cone-cylinder cathode can give electron emission planes around 360 degrees, thus larger electron emitting area is utilized. Secondly, the electrostatic field on the surface of a CNT cold-cathode not only is more uniform but also is enhanced. The reason of electrostatic field enhancement is that electrostatic field is compressed on surface of inner conductor in the coaxial electron gun. For example, when inner and outer radii are 1 mm and 5 mm, respectively, the electrostatic field on inner conductor surface is about 2.5 times stronger than that in an infinite plate with same potential difference (see supplementary Fig. s2 for details). In such cases, the electrostatic field along radial direction, which is needed for induced electron emission from CNTs, will be decreased, thus the occurrence probability of electric arc may be reduced under high voltage operation. Thirdly, because of crossed electric and magnetic fields, field emission electron beam will not reach the anode, so the transmission efficiency of electron beam can achieve 100% theoretically. Fourthly, through extending the axial length of CNT cold-cathode or enlarging the radius of inner conductor, the field emission area of cold-cathode can be increased to increase field emission current. Finally, electron beam current density can be increased more than 100 times when entering interaction cavity, thus field emission electron beam current density of CNT cold-cathode will be improved much more in those gun. As shown in Fig. 2a, electron beam is firstly compressed from the axial side of inner conductor to the radial section, and then will be accelerated by the anode and come into the beam tunnel. Here, electron beam will be secondly compressed on the radial direction with the magnetic field gradually increase, when the electron beam passes through the beam tunnel and moves into an interaction cavity.

This is the first time one has tried with such a design of electron gun using CNT cold-cathode, and we can show that it has high performance during our investigation of the terahertz gyrotron. Experiments were performed by using negative voltage pulses with duration of 100 μs. The characteristics are shown in Fig. 2b to 2e. Figure 2b shows that the maximum electron beam current reached 28.2 mA. The electron beam spot image is annular, as shown in Fig. 2c. The size of the electron beam can be obtained by comparing the beam spot size with the size of beam channel where electron goes through as marked up in Fig. 2c, and the outer radius of the electron beam is 1.276 mm and the inner radius 1.125 mmm. The compression ratio of electron beam is approximately 100 in our present design. The annular electron beam is nearly uniform on current density according to the luminance signal in the beam spot image. To evaluate the emission uniformity of the ring type beam spot, we captured the emission image of the ring spot, then selected 50 points along the ring, and measured their grayscales to represent the brightness intensity. The brightness intensity distribution along the ring spot is shown in the histogram in Fig. 2d. The uniformity of brightness distribution is calculated by the equation,


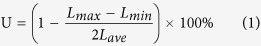


where L_max_ represents the maximum brightness measured, L_min_ represents the minimum brightness, L_ave_ represents the average brightness of the 50 points. Here the brightness uniformity of the ring spot is 75% which is acceptable for the application. The emission brightness stability in 14 minutes is also measured in the same way. The result in Fig. 2e shows that the brightness stability is good; the emission fluctuation of brightness is only 5.9%.

### Preparation of the curved-shape CNT-stainless steel cold-cathode and the physical property and field emission characteristics of the CNTs

To ensure the success of utilizing such a design of electron gun, we should have a high current with high current-density, uniformly emitting, large-area CNT cold-cathode. The particular difficulty is that the large-area substrate surface is curved (ie cylindrical stainless steel). In addition, such a surface is not smooth as a silicon one often used; deposition of catalyst on such a surface is not easy. We have tried to employ printing technique with CNT pasts^14^,^15^,^16^, and no satisfactory results were obtained due to not enough precise control on the thickness and the uniform distribution of CNTs. We then changed to further develop the method we invented early^17^, ie to grow CNTs on stainless steel substrate by using simple thermal vapor deposition method. Stainless steel contains iron and nickel, which can act as the catalyst for the growth of CNT. Firstly, we modify the preparation step, and develop a pretreatment technique for the selected kind of stainless steel material so that a uniform and nano-size catalyst layer may be formed. Secondly, we achieve uniform growth of CNTs on the cylindrical cone shape surface by special arrangement of sample within thermal CVD deposition tube. Thirdly, we refine growth conditions to ensure the CNT having high crystallizability and having no amorphous carbon layer in the interface between CNT and substrate. As a result, we found that our CNTs grow directly from substrate material. The details from our results are presented below to prove our points above.

The optimal growth process was carried out for a cone-cylinder 304 stainless steel electrode. Firstly, the substrate was cleaned ultrasonically by acetone, alcohol and deionized water respectively, dried by nitrogen gas flow to avoid pollutants. Then the cone-cylinder stainless steel substrate was putting in to a quartz tube in the CVD furnace. Special attention has to be paid to ensure that the sample size (including diameter and thickness) should be very small in comparison with those of quartz tube, so that the uniformity of thermal field distribution around the sample is not disturbed obviously. This is important to guarantee the uniform growth of CNTs on the curved-surface of stainless steel cathode. The furnace was heated to 850 °C under the protection of argon, and then injecting hydrogen for one hour to form catalyst particles. A mixed gas of hydrogen (H_2_) and ethylene (C_2_H_4_) was introduced to grow CNT films for 20 minutes. The hydrogen and ethylene flow rates were typically set at 200:20 SCCM. We use ethylene rather than acetylene and thus the growth temperature raises more than 100 °C. These changes result in high quality CNTs, as we have found. For hydrogen is a product of the hydrocarbon decomposition, hydrogen is co-flowed to systematically achieve the suitable balance for synthesis by neutralizing excess reactive carbon species and opposing the decomposition of the hydrocarbon feedstock. Also, hydrogen makes a cleaner product, with very little amorphous carbon present. Finally the substrate cooled down to room temperature under the protection of argon.

**Figure 3:**
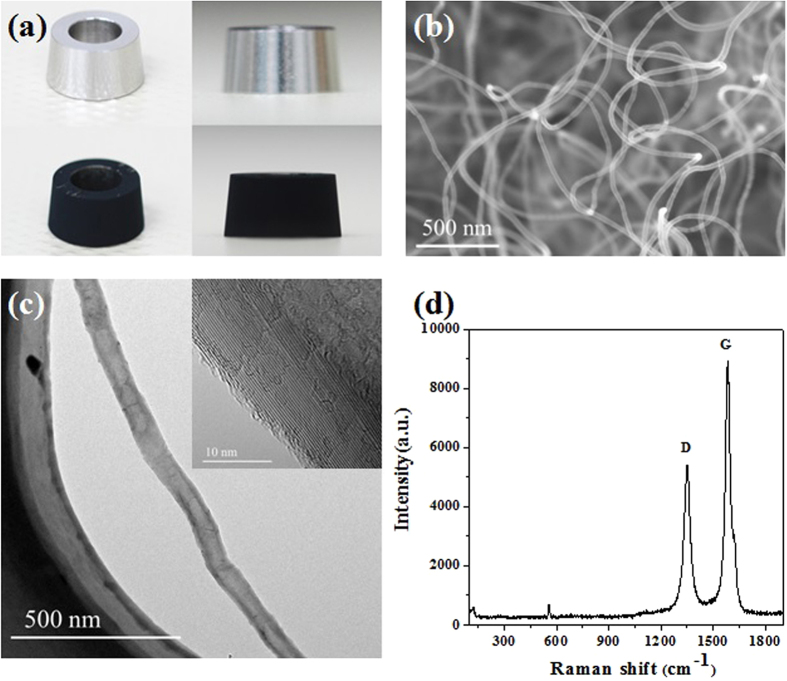
An image of the structure and morphology of the curved-shape CNT-SS cathode, where (**a**) top: before CNTs growth, and bottom: after CNTs growth, and (**b**) SEM image of CNTs morphology, and (**c**) TEM image of a typical CNT, the insert is the high resolution TEM image showing the graphite layer of CNT, and (**d**) Raman spectrum of CNTs grown on stainless steel. The D-band intensity is almost half of G-band feature, proving high structural quality of the sample.

The CNTs film covers uniformly the whole surface of cone-cylinder cathode electrode with large density, as shown in Fig. 3a. Typically, the height of cathode is 5.0 mm and the top and bottom diameter are 8.5 mm and 9.5 mm, respectively, and thus CNT-covered surface area is 1.41 cm^2^. The CNTs grow upward with a disorder shape (Fig. 3b), and their diameter typically ranges between 20–50 nm and the length is in between 2–6 μm, see Table 1. The TEM image (Fig. 3c) shows that a typical CNT has a smooth surface without amorphous carbon layer which demonstrates a good crystallization. The HRTEM image (Fig. 3c) shows the enlarged detail of the CNT walls that have about 36 graphite layers parallel to each other showing high quality with good graphitization, which means excellent electron transport ability and enables the high current carrying capacity. Typical Raman spectrum of the as-grown CNTs is shown in Fig. 3d. The ratio of I_D_/I_G_(ratio of G band over D band) results to be 0.61, about half of the G-band feature, suggesting that our carbon nanotubes are characterized by very few structural imperfections.

**Figure 4:**
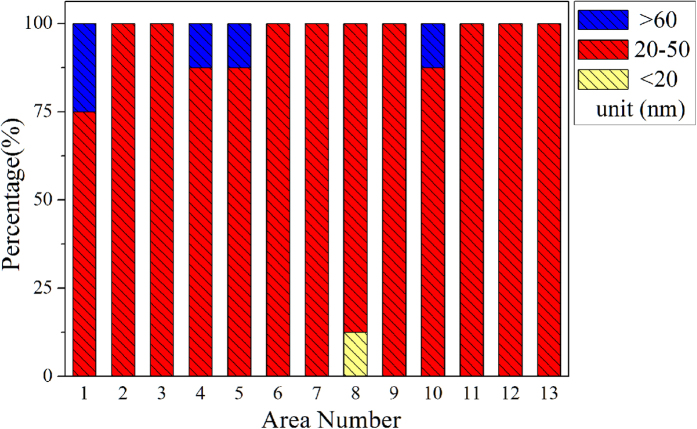
Totally the CNT diameter distribution of 13 different areas measured on the cone-cylinder cold-cathode.

To show that the CNTs are distributed on the whole substrate surface with similar geometrical parameters, the distribution of CNTs is statistically analyzed from 13 different areas selected from different angular position of the cone-cylinder cold-cathode, and totally the diameter of 104 CNTs are summarized, and shown in Fig. 4.

**Figure 5:**
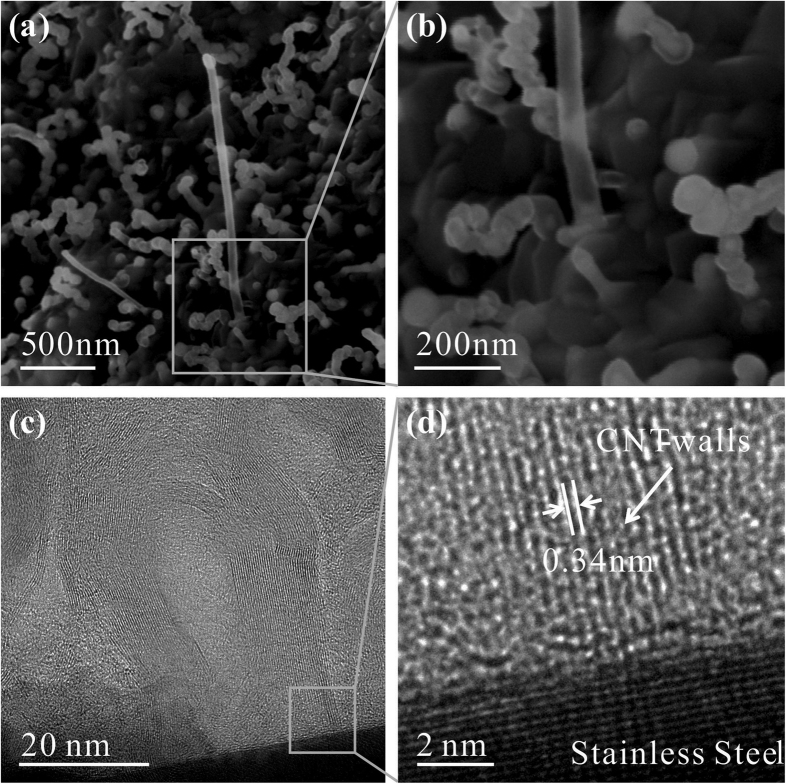
Cross-sectional SEM and TEM micrographs of the CNT cold-cathode showing the overview of the interfacial structure, where (**a**) SEM image and (**b**) high magnitude image of an initially-grown CNT directly connects with the substrate, (**c**) TEM image and (**d**) HRTEM image of one CNT grows from the stainless steel.

To answer why the individual CNT can sustain very high current and uniform emission over large area, we also studied the interface between CNTs and stainless steel substrate by SEM and TEM. The interface, full of importance, relates to the contact resistance and adherence of the nanotubes to the substrate and is a weak point where destructive vacuum breakdown may initiate^18^. After a long period time of growth, the CNT films often cover on the whole stainless steel. Not likes a silicon substrate which can be easily broken by a force at the edge, the stainless steel is difficult to be cut for observing the cross section of interface. To solve such a problem, we prepared samples with short growth time while keeping the other conditions same, so that the sample can be sectioned using the method developed by J B Park^19^. The CNTs were found being of around a micrometer long (Fig. 5a,b). The cross-section specimen was put into the TEM chamber for observation at 80 kV. We can see that the bottom of a CNT directly contacts with the stainless steel substrate without amorphous carbon layer (Fig. 5c). The high resolution TEM (HRTEM) shows the CNT walls are parallel to each other with a spacing of 0.34 nm and perpendicular to the substrate (Fig. 5d). Such near ideal contact interface between CNT and substrate is not seen before. Thus the CNTs have excellent electrical and mechanical contact, able to deliver large electron current from and dissipate heat to substrate under high-density large emission current operation.

**Figure 6:**
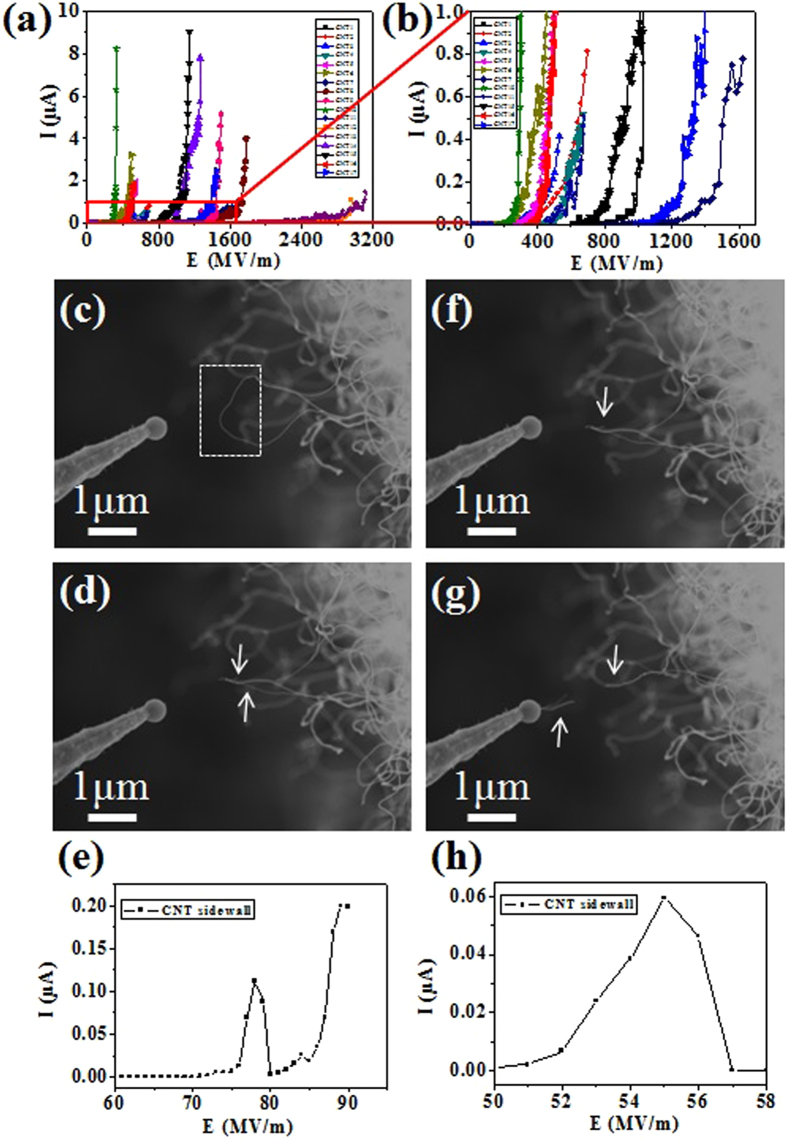
The field emission properties of the individual CNTs, where (**a**) I-E curve image from the individual CNT tips and (**b**) enlarged part indicated by red rectangle. (**c**) SEM image of the curved CNTs sidewall emitter with a diameter of 33 nm, (**d**) SEM image of a new emitter formed two CNTs, (**e**) the I-E curve during the above process occurred. (**f**,**g**) SEM images showing continued testing of the new emitter before and after the breakdown event, (**h**) the corresponding I-E curve recorded during the test process.

To investigate the emission capability, we study the possible emission sites and their emission performance. The field emission measurements on individual CNT tip and the curved sidewall of a single CNT are performed in a SEM chamber (ZEISS-Supra 55) equipped with a nano-manipulator, which is fixed with a tungsten tip. In the experiment, the CNTs be tested are as far away from the other CNTs as possible to exclude their interference. The distance between the anode probe and the tip of CNTs was typically set to around 1 μm. A picoammeter with a power supply (Keithley 6487) was employed to record the field emission current. The typical vacuum chamber pressure was ~5 × 10^−4 ^Pa. Field emission from sidewall of CNTs has not been paid enough attention to so far. The anode is driven positively using a variable DC voltage power supply. The field emission properties of the individual CNTs are given in Fig. 6. The maximum emission current from single CNT tips before vacuum breakdown ranges from hundreds of nanoamps to several microamps, as Fig. 6a,b show. We also find that there is large probability of electron emission from the sidewalls of CNTs. Figure 6c,d are SEM image of the CNT sidewall emitter with a diameter of 33 nm before and after the vacuum breakdown happened, forming a CNT bundle with two tips. The I-E curve (Fig. 6e) shows that the emission current reached 0.12 μA under the applied field of around 78 MV/m. Also, after the local vacuum breakdown, the CNT only broke locally and became a new emitter formed by two CNTs (Fig. 6d). Figure 6f,g show continued testing of the emitter, and it can reach the current of 0.06 μA (Fig. 6h) before another a local breakdown occurred, after which one CNT still remained. The experimental phenomena above prove that the curved sidewalls of CNTs make up an important part of electron emission sites, in addition to that from the CNTs tips. They enhanced significantly the emission capability of our CNT cold-cathode. We attribute this effect to direct benefits from the defect region in a single nanotube and the direct growth from stainless steel surface, otherwise local breakdown will cause destructive vacuum breakdown to the CNT cold-cathode, as described in our early work^18^. This new findings change the present comment view, ie field emission sites are from the tips of CNTs. Our finding reveals that field emission sites consist of two parts, tips and sidewalls of CNTs.

## Discussion

We demonstrate in this work that one can realize a cold-cathode terahertz-gyrotron using CNTs as field electron emitters. Our results move the frequency range from gigahertz region to terahertz region of cold-cathode high-frequency radiation sources. We can see that in principle there is no major difficulty in our cold-cathode gyrotron design to further increase output radiation frequency to higher end of terahertz range, since electrons in the vacuum device can move freely, ie not like in solid-state devices. In addition, a maximum beam current of 28.2 mA, and the beam current density of 2.477 A/cm^2^ and an average beam radius of 1.276 mm are achieved on the output port of this cold cathode electron gun, which the electron beam is circular. The results indicate that higher output power and higher radiation frequency terahertz gyrotron may be made using CNT cold-cathode electron gun. By further optimizing the CNT cold-cathode electron gun design and performance, significant larger current and current density, better focusing property and electron beam injection capability may be obtained, and a lot higher output power of terahertz radiation may be expected. In fact, the realization of a new design is in the process. Finally, the success of the all of above relies on the capability of unique preparation technique of CNTs on curved stainless-steel substrate, i.e. the curved-shape CNT-stainless steel cold-cathode (curved-shape CNT-SS cathode). We have developed a number of unique techniques and they may be further improved. We also have two important scientific findings: a) there is large probability of electron emission from the curved sidewall of CNT in addition to that from the tips, leading to greatly enhanced emitting capability, and b) CNTs can grow directly from stainless-steel without other barrier layer, thus avoiding electrical breakdown in the interface between CNT and substrate. These developments and findings enable one using conventional magnetic injection electron gun, so that a large area emitting surface is utilized to deliver large current for electron beam. One can further study the underlying scientific principles of such findings, which may lead to significantly enhanced performance of the curved-shape CNT-SS cathode.

## Method

### The characterization of gyrotron

The CNT cold-cathode gyrotron experimental system is shown in supplementary Fig. s1. The CNT cold-cathode gyrotron is set into a super conducting magnet system. A negative high voltage power supply with a pulse duration of 100 μs is used to test the output signal of CNT cold-cathode gyrotron. The maximum output voltage of negative high voltage power supply is −70 kV. High voltage is divided by resistances R_1_ and R_2_ (R_2_:R_1_ = 2:1) to provide negative high voltage for the CNT cold cathode and control anode. The anode is connected to the earth. The CH_1_ channel of oscilloscope is detected the high voltage signal by a non-inductive resistor R_0_ (R_0_ << R_1_ + R_2_). The CH_2_ channel of oscilloscope is tested the output signal of CNT cold-cathode gyrotron by frequency and power detectors.

## Additional Information

**How to cite this article**: Yuan, X. *et al*. A Fully-Sealed Carbon-Nanotube Cold-Cathode Terahertz Gyrotron. *Sci. Rep.* **6**, 32936; doi: 10.1038/srep32936 (2016).

## Supplementary Material

Supplementary Information

## Figures and Tables

**Table 1:** Diameter distribution of CNTs from 13 different areas of the cone-cylinder cold-cathode.

Diameter distribution of CNT						
Diameter (nm)	<20	20–30	30–40	40–50	50–60	>60
Number	1	30	48	20	4	1
Percentage (%)	1.0	28.8	46.2	19.2	3.8	1.0
