# Supplementary material for: A Fully-Sealed Carbon-Nanotube Cold-Cathode Terahertz Gyrotron
Source: Sci Rep. 2016 Sep 9;6:32936. doi: 10.1038/srep32936 (PMC5017026; doi:10.1038/srep32936)
Supplement: Supplementary Information [file srep32936-s1.pdf]

Supporting Information

A Fully-Sealed Carbon-Nanotube Cold-Cathode Terahertz Gyrotron

Xuesong Yuan[b], Weiwei Zhu[a], Yu Zhang[a], Ningsheng Xu[a] ,

Yang Yan[b], Jianqiang Wu[b], Yan Shen[a], Jun Chen[a], Juncong She[a], Shaozhi Deng[a*]

[a]State Key Laboratory Optoelectronic Materials and Technologies, Guangdong Province Key Laboratory of Display Material and Technology, and School of Electronics and Information Technology, Sun Yat-Sen University, Guangzhou, China, 510275

[b]School of Physical Electronics,University of Electronic Science and Technology of China, Chengdu, China, 610054

*Corresponding author email: stsdsz@mail.sysu.edu.cn

The characterization of gyrotron

Fig.s1 shows the CNT cold-cathode gyrotron experimental system. The CNT cold-cathode gyrotron is set into a super conducting magnet system. A negative high voltage pulse power supply is used to test the output signal of CNT cold-cathode gyrotron. The high voltage is divided by resistances R_1 and R_2 to provide negative high voltage for the CNT cold cathode and control anode. The anode is connected to the earth. The CH_1 channel of oscilloscope is detected the high voltage signal by a non-inductive resistor R_0 ($R_0 << R_1 + R_2$). The CH_2 channel of oscilloscope is tested the output signal of CNT cold-cathode gyrotron by frequency and power detectors.

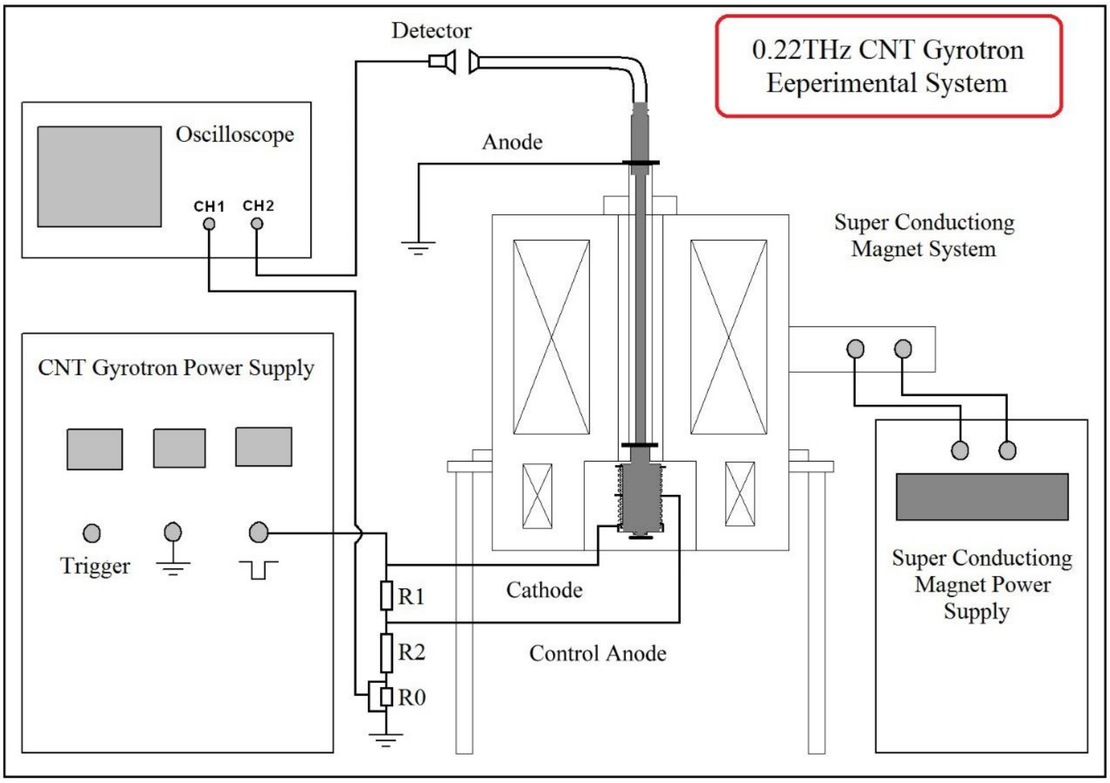

Fig.s1 Block diagram of 0.22THz carbon-nanotube cold-cathode gyrotron experimental system.

The electrostatic field simulations and calculations for the coaxial cylinder cathode

Fig. s2 shows the electric line of force in infinite plate model and coaxial model, and simulation results of electrostatic field on cross-section of coaxial model. In the infinite plate model, the electric line of force is uniform distribution between two plates and the electrostatic field can be obtained by the equation: $E=U/d$. Where U is the voltage difference between two plates, d is the distance between two plates. In the coaxial model, the electric line of force will be compressed on surface of inner conductor. Thus the electrostatic field will be enhanced. The calculation equation of electrostatic field is $E = U/R\ln(R_o/R_i)$. Where R is radial position, R_i and R_o are inner and outer radii of coaxial model, respectively. When R_i =1 mm, R_o=5 mm, d=R_o-R_i=4 mm and U=4 kV. The electrostatic field on inner conductor surface is 2.485 kV/mm and the electrostatic field on outer conductor surface is 0.497 kV/mm, Figure 1(c) shows the simulation results. However, the electrostatic field is 1 kV/mm in the infinite plate

model. Thus the electrostatic field on inner conductor surface is about 2.5 times stronger than that in an infinite plate with same potential difference.

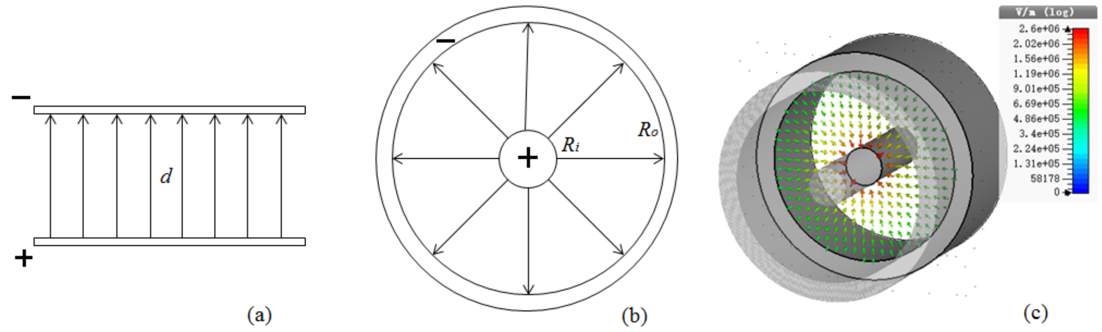

Fig. s2 (a) Electric line of force distribution in infinite plate model. (b) Electric line of force distribution in coaxial model. (c)Electrostatic field on cross-section of coaxial model
